# Evaluation of the Potential Diagnostic Utility of the Determination of Selected Immunological and Molecular Parameters in Patients with Ovarian Cancer

**DOI:** 10.3390/diagnostics13101714

**Published:** 2023-05-12

**Authors:** Aleksandra Englisz, Marta Smycz-Kubańska, Aleksandra Mielczarek-Palacz

**Affiliations:** 1 The Doctoral School, Medical University of Silesia, 40-055 Katowice, Poland; 2 Department of Immunology and Serology, Faculty of Pharmaceutical Sciences in Sosnowiec, Medical University of Silesia, 40-055 Katowice, Poland

**Keywords:** ovarian cancer, potential biomarkers, galectins, adipocytokines, miRNA

## Abstract

Ovarian cancer is one of the most serious challenges in modern gynaecological oncology. Due to its non-specific symptoms and the lack of an effective screening procedure to detect the disease at an early stage, ovarian cancer is still marked by a high mortality rate among women. For this reason, a great deal of research is being carried out to find new markers that can be used in the detection of ovarian cancer to improve early diagnosis and survival rates of women with ovarian cancer. Our study focuses on presenting the currently used diagnostic markers and the latest selected immunological and molecular parameters being currently investigated for their potential use in the development of new diagnostic and therapeutic strategies.

## 1. Introductions

Ovarian cancer represents a heterogeneous group of cancers characterised by distinct risk factors, precursor lesions, pathogenesis, pattern of spread, molecular profiles, clinical course and response to chemotherapy [1]. The 2020 World Health Organisation (WHO) classification of female genital cancers, based on histopathology, molecular analysis and immunoprofiling, distinguishes at least five main types of ovarian cancer: high-grade serous carcinoma (HGSC, 70%), endometrioid carcinoma (EC, 10%), clear cell carcinoma (CCC, 6–10%), low-grade serous carcinoma (LGSC, 5%) and mucinous carcinoma (MC, 3–4%) [1]. In 2004, Shih and Kurman [2] proposed a division of ovarian cancers into two types: type I and type II. Type I tumours include well-differentiated serous (LGSC), endometrioid, clear cell and mucinous carcinomas, characterised by a slow growth rate and little tendency to spread. Type I cancers are usually confined to the ovaries (stage I) and have a favourable prognosis, accounting for 10% of ovarian cancer deaths. Type II cancers include low-differentiated serous carcinomas, low-differentiated endometrioid carcinomas, sarcomas and undifferentiated carcinomas, characterised by a very rapid growth rate and high aggressiveness. In 75% of cases, they are diagnosed at an advanced stage (FIGO III–IV), which, in consequence, entails a poor prognosis. Type II tumours are responsible for 90% of deaths from ovarian cancer [2,3,4].

Ovarian cancer is often characterised by an asymptomatic course in the early stages, which contributes to the late detection of lesions and high mortality rates in women. The symptoms appear in the late stages of the disease and are non-specific, e.g., bloating, abdominal pain, back pain, indigestion or feeling of early satiety [5]. Most cases of ovarian cancer are diagnosed at stage III and IV of the disease, when the five-year survival rate is less than 30% [6]. A comparison of the characteristics of type I and type II ovarian cancer is presented in Table 1.

To date, no effective screening procedure has been established to detect the disease at an early stage. Therefore, tumour markers that could be used to diagnose ovarian cancer at an early stage are being intensively searched for. 

An ovarian cancer diagnosis includes gynaecological examination, imaging tests such as ultrasonography (USG), computed tomography (CT), magnetic resonance imaging (MRI) and measurement of tumour marker concentrations, the most common of which are CA125 and HE-4 [8]. 

This study aims to gather up-to-date information on currently used markers in the diagnosis of ovarian cancer, as well as immunological and molecular parameters currently the subject of numerous studies aimed at developing new diagnostic and therapeutic regimens.

## 2. Group Markers Used in the Diagnosis of Ovarian Cancer

### 2.1. CA125

The biomarker most commonly used in the diagnosis of ovarian cancer is tumour antigen 125 (carbohydrate antigen 125, CA125), first described in 1981 by Bast et al. [9]. This protein was determined at elevated serum levels in more than 80% of women diagnosed with ovarian cancer. An increase or decrease in CA125 levels correlated with disease progression or regression in 93% of cases, which suggested that CA125 might be helpful in monitoring response to treatment in patients with epithelial ovarian cancer [9,10]. This antigen has played the most significant role in the screening, detection and treatment of ovarian cancer over the past four decades [4]. The CA125 protein, encoded by the MUC16 gene, is one of the mucin-derived glycoproteins associated with the cell membrane of many epithelial cell types [8]. CA125 levels are elevated in only about half of cases of stage I ovarian cancer (mainly type I) and in 92% of cases of advanced forms of the disease (mainly type II) [4,11]. Elevated CA125 levels also occur in other cancers, including endometrial, breast, pancreatic, gastrointestinal and lung cancers. Elevated levels of the biomarker may occur in normal menstruation, pregnancy, endometriosis and even in non-gynaecological diseases such as inflammatory conditions of the liver and pancreas [6,11]. CA125 antigen has a too low sensitivity and specificity to be considered a reliable biomarker for the early detection of ovarian cancer [6] and therefore cannot be used as a single marker [12]. A similar opinion was expressed by Chen M. et al. [13]. In their opinion, CA125 testing in the blood is the most sensitive and specific marker available for the early detection of ovarian cancer. However, the method is still suboptimal due to its low sensitivity in early stages of the disease, and its predictive value for screening is limited [13].

New, more effective screening strategies are currently being explored. More than 110 potential protein biomarkers have been evaluated individually and in combination with CA125 [14]. 

The biomarkers CA125, HE4, apolipoprotein A1, transthyretin, transferrin and β2-macroglobulin have been introduced into the RMI, ROMA or OVA1 algorithms used to differentiate benign from malignant disease [15]. 

Yurkovetsky et al. [16] showed that a panel of four biomarkers consisting of CA125, HE4, CEA and VCAM-1 detected the early stage of ovarian cancer with 86% sensitivity and the later stages with 93% sensitivity at 98% specificity, using multiplex xMAP bead-based immunoassays. In the same study group, the single marker CA125 had a sensitivity of 61% for early-stage ovarian cancer and 83% for later stages [16]. Van Calster et al. [17] showed that approximately 75% of ovarian cancer patients had CA125 levels above 35 U/mL, while 60% of patients had levels above 100 U/mL [17]. Increasing the CA125 cut-off significantly increased its specificity at the expense of sensitivity [18]. According to Charkhchi et al. [4], diagnosis of type II ovarian cancer using CA125 may be possible with a higher cut-off point than 35 U/mL through the use of point-of-care CA125 assays in primary care facilities [4]. Lee M [19] et al. investigated the relationship of CA125 levels after the first cycle of chemotherapy and survival time of patients with advanced ovarian cancer. They showed that CA125 levels after the first cycle of chemotherapy were the most significant independent prognostic factor for overall survival (OS). Time to normalisation (*p* = 0.028) and the relative percentage change between baseline CA125 concentration and after the first cycle of chemotherapy (*p* = 0.021) were additional independent prognostic factors for OS. The CA125 concentration after the first cycle of chemotherapy (*p* = 0.001) and time to normalisation (*p* < 0.001) were found to be independent prognostic factors for progression-free survival (PFS) [19].

### 2.2. HE4

The HE4 (human epididymis protein 4) marker is a low-molecular-weight glycoprotein first identified by Kirchhoff et al. [20] in the epithelium of the distal epididymis as a protease inhibitor involved in sperm maturation [20]. HE4 is encoded by the WFDC2 gene, located at the locus of chromosome 20q12-13, the most frequently amplified genomic sequence in ovarian cancer, pointing to the role of this protein in tumourigenesis [21]. HE4 is not found in the ovarian surface epithelium but is overexpressed in ovarian cancer tissues, where it is secreted into the extracellular environment and therefore can be detected in serum [22]. Studies have shown the expression of HE4 in many tissues outside the male reproductive system, including regions of the respiratory tract and nasopharynx [23]. HE4 gene expression was highest in the normal human trachea and salivary gland, and to a lesser extent in the lungs, prostate, pituitary gland, thyroid and kidney. Among 175 cases of cancers in adults, the gene expression was highest in ovarian serous carcinomas [24]. Using a real-time quantitative PCR method in their studies, Li J. et al. [25] detected high levels of HE4 mRNA in the epididymis, trachea and lungs and medium levels in the prostate, endometrium and breasts. Low or no expression was detected in the colon, ovary, liver, placenta, peripheral blood cells and skeletal muscles [25]. There have been numerous studies using the HE4 marker in serum. Scalleta G. et al. [26] analysed the role of HE4 in the diagnosis, prognosis and monitoring of ovarian tumours. In their opinion, serum HE4 levels are a useful preoperative test for predicting the benign or malignant nature of pelvic tumours, and HE4 itself appears to better predict disease recurrence when compared to CA125 [26]. Moore G. et al. [27] in their study showed that elevated serum HE4 levels correlate with chemoresistance and reduced survival in patients with ovarian cancer [27]. HE4 showed better sensitivity and specificity in diagnosing ovarian cancer recurrence in relation to CA125, being at the same time an earlier indicator of disease recurrence, with a 5- to 8-month lead time. HE4 showed better performance when applied in combination with other markers (CA125, CA-72.4) [28]. A study by Janas Ł et al. [29] shows that HE4 concentrations, unlike serum CA125 concentrations, are not elevated in patients with endometriosis, and that HE4 increases diagnostic efficiency by verifying false-positive CA125 results [29]. Moore et al. [30] showed that HE4 levels were elevated in more than 50% of ovarian tumours where CA125 levels remained stable. HE4 was shown to have a higher sensitivity in patients with early-stage disease [30]. Han L. N. et al. [31] conducted a meta-analysis of 38 published studies evaluating the association between HE4 and prognosis in endometrial cancer (EC). They found that high serum HE4 levels were associated with FIGO stages III–IV as well as with poor overall survival and disease-free survival. Therefore, when compared with CA125, the serum HE4 marker may be a better prognostic indicator for EC [31].

### 2.3. Mesothelin

Mesothelin is a tumour differentiation antigen present on pleural, peritoneal and pericardial mesothelial cells [32]. The coding gene is located on the short arm of chromosome 16. Mesothelin levels can be determined in blood serum, peritoneal fluid and urine [33]. In normal mesothelial cells, this marker is present in trace amounts, whereas mesothelin levels are high in *mesothelioma*, pancreatic cancer and ovarian cancer (approximately 70% of cases) [34]. McIntosh M. W. et al. [35] evaluated the usefulness of a composite marker (CM), a combination of CA125 and SMR (soluble mesothelin related), in sera from 52 women with ovarian cancer, 43 women with benign ovarian tumours and 220 healthy women. The sensitivity, specificity and temporal stability of CA125, SMR and the combined marker (CA125 and SMR) were assessed. The results showed that at 98% specificity, the CM identifies 86.5% (45 of 52) of the cases [35]. Badgwell D. et al. [36], analysed mesothelin concentrations in the serum and urine of women. Urinary mesothelin levels were shown to be a better indicator of ovarian cancer than serum levels, particularly in early forms (FIGO I and II). Elevated serum mesothelin values were observed in 12% of women with early-stage lesions (FIGO I, II) and in 48% of women with advanced stages of ovarian cancer (FIGO III and IV), with a test specificity of 95%. Urine analysis of women with early stages of ovarian cancer showed increased levels of mesothelin in 42% of cases and in 75% of women with advanced stages of cancer. This biomarker has a too low sensitivity for use in screening, but it has been suggested that the determination of mesothelin simultaneously with other markers could increase the sensitivity of detection of ovarian cancer in early stages [36]. While reviewing studies, Giordano G. et al. [37] noted that many of them suggest that combining mesothelin-targeted therapies with other chemotherapeutics may yield satisfactory results, achieving a significant response and prolonging overall survival in patients with HGSOC [37]. Li Y et al. [38] detected mesothelin overexpression in ovarian cancer. They confirmed MSLN overexpression in chemoresistant ovarian cancer cell lines. Their study suggests that MSLN is involved in various pathways involved in suppressing immune activation and promoting chemoresistance, leading to a poor prognosis in ovarian cancer [38]. It has been suggested that the determination of mesothelin simultaneously with other markers could increase the sensitivity of detecting ovarian cancer at early stages [8]. As a single marker, mesothelin does not apply to the diagnosis of ovarian cancer, but it could complement standard diagnostic methods based on ultrasound and currently used tests or algorithms (CA125, OVA1, ROMA) and significantly improve their sensitivity [33].

### 2.4. Osteopontin

Osteopontin (OPN) is an extracellular matrix glycoprotein involved in many cellular processes, including wound healing, inflammation, immune response and tumourigenesis [39]. Osteopontin is expressed not only in ovarian cancer, but also in endometrial cancer, cervical cancer, breast cancer, colorectal cancer, non-small cell lung cancer, prostate cancer, hepatocellular carcinoma and gastric cancer. OPN is associated with tumour progression, invasion and metastasis [6]. In the diagnosis of ovarian cancer, osteopontin has been intensively studied, as evidenced by numerous meta-analyses. Lan Z. et al. [40] conducted a meta-analysis of studies which evaluated the role of osteopontin alone and its combination with CA125 in the diagnosis of ovarian tumours. They showed that the diagnostic sensitivity and specificity for osteopontin in ovarian cancer were 0.766 (95% CI 0.685–0.831) and 0.897 (95% CI 0.849–0.931), respectively. In contrast, for osteopontin and CA125, the sensitivity and specificity amounted to 0.871 (95% CI 0.788–0.924) and 0.881 (95% CI 0.837–0.914), respectively. It is suggested that osteopontin is a useful biomarker for ovarian cancer screening and may be a promising complementary test to CA125. However, according to the authors, it still requires additional research [40]. Another meta-analysis was conducted by Wang Y. D. et al. [41], the results of which showed a positive association between serum OPN levels and ovarian cancer (SMD = 2.60, 95% CI 1.88–3.32, *p* < 0.001). It was therefore concluded that serum OPN levels may be useful in the diagnosis of ovarian cancer [41]. High levels of osteopontin were reported in urine samples of patients with high-grade ovarian cancer, suggesting that the test could potentially be used as a non-invasive tool for the early diagnosis of ovarian cancer [42]. 

### 2.5. CA72-4

CA72-4 is a tumour-associated glycoprotein. It represents a distinct epitope on the mucin MUC1, and its elevated levels have been detected in ovarian cancer. Its levels are not affected by pregnancy, the menstrual cycle or endometriosis and are only slightly affected by inflammation [43]. There have been many reports on the utility of CA72-4 in combination with other biomarkers. Wang Q. et al. [44] evaluated the diagnostic utility of HE4, CA125, ROMA index and CA72-4 in the diagnosis of ovarian cancer. The HE4 marker had the best specificity, CA72-4 had the lowest sensitivity, and the ROMA index had the best diagnostic efficiency among the biomarkers tested in the diagnosis of ovarian cancer. In contrast, CA125 had better specificity in postmenopausal patients [44]. Li M. et al. [45] evaluated the usefulness of CA72-4 and CA15-3 among three groups of patients: those diagnosed with ovarian cancer, with cervical cancer and with endometrial cancer. Concentrations of both parameters were significantly higher in all three groups of patients. There was no difference in CA72-4 levels between the groups [45].

### 2.6. Transthyretin (TTR)

Transthyretin (TTR) is a protein involved in the transport of thyroid hormones in the blood and plays an important role in retinol metabolism. TTR levels have been found to be reduced in the serum of patients with ovarian cancer and advanced cervical and endometrial cancer [46]. The results of Zheng X. et al. [47] showed that TTR is a better marker for detecting early-stage ovarian cancer (stages I and II) than CA125 and HE4 [47]. Kozak et al. [48] combined the determination of TTR with CA125, haemoglobin (Hb), apolipoprotein AI and transferrin (TR). According to the researchers, this combination may be beneficial in detecting early-stage ovarian cancer [48]. Kim Y.W. et al. [49] showed a significant improvement in sensitivity in the diagnosis of ovarian cancer using a combination of three serum biomarkers: CA125, transthyretin and apolipoprotein A1 (ApoAI). The combination of transthyretin and apolipoprotein A1 with CA125 improved both the sensitivity and specificity of ovarian cancer diagnosis compared with the sensitivity and specificity of the individual biomarkers determined separately [49].

The most commonly used biomarker to date, CA125, as well as the other discussed tests, combinations and algorithms, do not yet meet the criteria necessary for the early detection of ovarian cancer. There is still a continuing need for further research to find new approaches which would be applicable in the diagnosis of ovarian cancer. The present study provides up-to-date information on new immunological and molecular parameters, such as galectins, cancer stem cells, microRNA molecules, autoantibodies, circulating DNA molecules or adipocytokines, which may find a possible future application in the development of new diagnostic and prognostic schemes in ovarian cancer patients.

## 3. Group Algorithms in the Diagnosis of Ovarian Cancer

### 3.1. ROMA Algorithm

The Risk of Ovarian Malignancy Algorithm (ROMA) is a tool recognised in 2011 by the US Food and Drug Administration (FDA) for assessing the risk of ovarian malignancy. The ROMA algorithm comprises determination of serum concentrations of two tumour markers—CA125 and HE4—and a statistical estimation of the risk of a malignant ovarian tumour, taking into account the patient’s menopausal status. The result of the algorithm is given as a percentage indicating the probability of developing a malignancy [50]. A number of studies have shown that simultaneous assessment of HE4 and CA125 protein levels with ROMA calculation is useful in the diagnosis of ovarian cancer [51]. 

Baar C. E. et al. [52] found that combining the HE4 assay with the currently used CA125 test as part of the ROMA algorithm improved the detection of ovarian cancer in primary care, particularly in women under 50 years of age, when the diagnosis is more difficult. However, these results need to be validated in a larger trial [52]. According to a study by Dochez V. et al. [53] on the efficacy of HE4, CA125 assays, RMI and ROMA algorithms, the most effective diagnostic tool for ovarian cancer diagnosis to date is the combination of CA125 and HE4 [53]. The Polish Gynaecological Society recommends the calculation of the RMI index and the ROMA test in all cases of ovarian tumours [54]. Braicu E. I. et al. [55] evaluated the diagnostic value of a panel of tests consisting of USG, HE4, CA125 and ROMA, both to detect ovarian cancer in patients over 18 years of age (*N* = 965) and to distinguish benign and malignant adnexal tumours. Of these patients, 804 were diagnosed with benign tumours and 161 with ovarian cancer. In stage I and II disease, the diagnostic efficacy of all three biomarkers was lower (AUC < 0.77) than in later stages (AUC > 0.92). In the differential diagnosis of ovarian cancer and endometriosis, the ROMA algorithm and the HE4 marker performed better than CA125 (sensitivity 99 and 98.1%, versus 75.0%, respectively) [55]. A similar assessment was performed by Shin K.H. et al. [56] evaluating the clinical utility of CA125, HE4 and CA72-4, and ROMA in the differential diagnosis of malignant and borderline tumours among women (*N* = 266; 213 benign tumours, 14 borderline tumours and 39 malignant tumours). All tested markers were significantly higher in the group of women with malignant tumours than in the group of women whose lesions were benign. The HE4 concentration and ROMA values were also significantly higher in the group of women with malignant tumours than in the group of women with borderline tumours. The ROMA test had the highest AUC value for distinguishing malignant and borderline tumours from benign tumours in premenopausal (0.773) and postmenopausal patients (0.927). CA125 levels were significantly higher in patients with endometriosis (*p* < 0.001), whereas HE4 and CA72-4 levels were not endometriosis-dependent (*p* = 0.128 and 0.271, respectively) [56].

### 3.2. RMI

The RMI (Risk of Malignancy Index) Ovarian Cancer Risk Index, proposed in 1990 by Jacobs et al. [57], takes into account the sonomorphological characteristics of the tumour (U), the patient’s age (premenopausal—1 point, postmenopausal—3 pts) and the serum CA125 antigen concentration in IU/mL. The formula used for the calculation is as follows: RMI = U × age × CA125 (IU/mL). In the study by Jacobs et al. [57], RMI was calculated for 101 patients with benign disease and 42 patients with malignant pelvic masses. Using an RMI threshold of 200, the sensitivity of the algorithm amounted to 85%, while the specificity was 97%. A score above 200 indicates a significantly increased risk of an ovarian malignant tumour [57]. Similar findings were presented by Al-Asadi J.W. et al. [58], who used RMI2 with a cut-off value of 200 to differentiate between benign and malignant tumours. The final diagnosis was confirmed with a histopathological examination. The sensitivity of the RMI was 100%, specificity 96.2%, positive predictive value 87.5% and the negative predictive value 100%. RMI identified malignant cases more accurately than any single criterion in the diagnosis of ovarian cancer [58].

### 3.3. OVA1 Test

The OVA1 test is an FDA (Food and Drug Administration)-approved assay, which determines plasma concentrations of five proteins associated with tumourigenesis (CA125, apolipoprotein A-1 (ApoA-1), transthyretin (TTR), transferrin (TF) and β2-microglobulin) to calculate an ovarian malignancy risk index score [32]. OVA1 provided 96% sensitivity with 28% specificity in postmenopausal women and 85% sensitivity with 40% specificity in premenopausal women [59]. The OVA1 test detects more early-stage malignancies compared to a single CA125 assay. By using only CA125 as a screening test, a proportion of cases would be missed. The use of OVA1 may lead to early detection and a better prognosis in ovarian cancer patients [60].

## 4. Group Potential Immunological and Molecular Parameters in the Diagnosis of Ovarian Cancer

### 4.1. Galectins

Galectins are a group of proteins from the lectin family, characterised by their ability to bind β-galactosides due to the presence of a carbohydrate recognition domain (CRD). Currently, 16 proteins belonging to galectins have been identified. These proteins are involved in both physiological processes, such as cell proliferation and apoptosis or the immune response, and pathological processes, such as tumour progression and metastasis [61,62,63]. The results of the studies to date suggest a complex role for these proteins in ovarian cancer carcinogenesis. The Gal-1, Gal-3, Gal-7, Gal-8 and Gal-9 galectins have been observed to play a role in ovarian cancer formation and progression. Galectins also appear to be a useful diagnostic and prognostic tool for assessing tumour progression or the efficacy of therapy in ovarian cancer patients, but this requires further research [61]. Gal 1, 3, 7 and 9 appear to have the greatest impact on the invasiveness, metastasis, chemoresistance and immunosuppressiveness of ovarian cancer [61]. Increased expression of galectin-1 in primary ovarian cancer tissue has been demonstrated, while it is absent in normal tissue. Galectin-1 is likely to enhance the proliferation and invasiveness of ovarian cancer cells, and its expression level is positively correlated with tumour stage and lower susceptibility to chemotherapy with cisplatin [64,65,66]. Gal-1 is thought to contribute to tumour transformation, cell cycle regulation, apoptosis, cell adhesion, migration and inflammation and may become a molecular target for the development of new therapeutic tools [67]. Abdelwahab M. et al. [68] evaluated serum galectin-1 levels and its expression in patients at different stages of serous ovarian cancer (SOC). They showed that serum galectin-1 level was significantly associated with FIGO stage (III, IV > I, II) [68]. Masoodi M. et al. [69] found that galectin-1 levels were elevated in epithelial ovarian tumours, with significantly higher levels in the serous subtype. After surgical intervention and chemotherapy, galectin-1 levels were found to decrease. The concentration of galectin-1 differed markedly in the serum of patients compared to the CA125 determinations [69]. Conflicting reports concern the evaluation of galectin-3 expression. Both significantly increased [70] and decreased [71] Gal-3 expression relative to that in normal tissue has been demonstrated. A lack of association between increased galectin-3 levels and patient age, stage and histological type of tumour was observed, while a positive correlation between the expression of only the cytoplasmic fraction of galectin-3 and a poor prognosis was also observed [70]. It has been suggested that galectin-3 expression in ovarian cancer probably depends on tumour- or tissue-specific factors that may modulate its levels, which requires further study [66,71]. In contrast, in benign and malignant ovarian tumours, an increase in galectin-7 expression has been shown, while no galectin-7 expression has been reported in healthy ovarian tissue. Furthermore, an increase in galectin-7 expression has been shown to result in tumour progression through increased cancer cell invasiveness and a proapoptotic effect on immune cells [66,72]. Labrie M et al. [73] showed that high plasma levels of gal-8 and gal-9 were associated with lower OS and PFS. They observed that Gal-8 concentrations were significantly higher in the plasma of patients with high-grade serous carcinoma compared to the plasma of healthy women [73]. Shultz et al. [74] showed that Gal-8 expression was a positive prognostic factor for overall and disease-free survival of ovarian cancer patients. In the case of galectin-9, its moderate expression correlated with shortened survival, while high Gal-9 expression indicated more favourable OS and PFS [74]. Mohamed RM. et al. [63] conducted a study evaluating the mRNA expression of galectins-1, -3, -4, -8 and -9 in epithelial ovarian cancers with qRT-PCR. *LGALS1*, *LGALS3*, *LGALS4*, *LGALS8* and *LGALS9* were found to be overexpressed in ovarian cancer patients. The expression of galectin-3 and -9 was found to be significantly elevated in lymph node metastases (p = 0.044 and p = 0.011). The observed increase in galectin-1 and -9 expression was statistically significant in stages IIB, IIC and IIIB (p = 0.002) according to the FIGO classification. These results support a role for galectins in carcinogenesis, disease progression and lymph node metastasis in ovarian cancer [63]. Summary information on selected galectins is presented in Table 2.

### 4.2. Adipocytokines

Adipocytokines are biologically active proteins with multidirectional effects, produced and secreted by adipose tissue [77], exhibiting autocrine, paracrine and endocrine effects [78]. Many of them have hormonal properties, e.g., leptin, adiponectin, resistin, visfatin, apelin, chemerin, and omentin. Excess adipose tissue accompanying obesity contributes to the enhancement of inflammation, which may be responsible for the higher incidence of cancer in obese patients [79]. The relationship between obesity (expressed, inter alia, by BMI) and ovarian cancer risk has not yet been sufficiently investigated. Few studies describe the association of elevated BMI with an increased risk of invasive endometroid cancer, mucinous carcinoma, clear cell carcinoma or LGSOC [80]. The present study characterises selected adipocytokines and their potential role in ovarian cancer. 

#### 4.2.1. Leptin

Leptin is a protein with multidirectional actions—neurohormonal, metabolic and immunomodulatory [77]—formed mainly in mature white adipose tissue cells, the biosynthesis and secretion of which are dependent on adipose tissue mass [81]. This protein is involved in the regulation of appetite and metabolism and has a stimulatory effect on the macrophage function. Leptin concentration increases at the site of inflammation after IL-1β stimulation [80]. Signalling pathways mediated by leptin play an important role in tumour cell proliferation, invasion and metastasis. Leptin exerts its action through the specific membrane receptor Ob-R (obesity receptor; class I cytokine receptor) [82]. The findings of Gu F. et al. [83] suggest that leptin has an important role in chemoresistance and may serve as a novel therapeutic target for ovarian cancer in patients treated with platinum and paclitaxel [83]. Leptin is known to activate a variety of signalling pathways, in particular the JAK2/STAT3 pathway included in these cancer cells. Kumar et al. [84] evaluated the role of leptin and its receptor LEPR using ovarian cancer cell lines. The study showed that leptin stimulation led to an increased proliferation, survival and migration of LEPR-expressing ovarian cancer cell lines, with these effects shown to be mediated by the JAK2/ STAT3 pathway [84]. Kim KT. et al. [85] demonstrated the expression of both leptin and its receptor in various types of ovarian cancer, except the mucinous type [85]. They also demonstrated the stimulatory effect of lectin on the growth of cancer cell cultures isolated from the ovary [80] and that overexpression of leptin and its receptor in ovarian cancer tissue indicates the aggressive nature of the disease [86]. Uddin S. et al. [82] analysed the role of leptin and its mechanisms of action in EOC tissue samples and cell lines and showed that leptin stimulates cell proliferation and inhibits apoptosis through activation of the PI3K/AKT signalling pathway [82]. 

#### 4.2.2. Resistin

Resistin is involved in the development of inflammation, regulating carbohydrate and lipid metabolism and stimulating endothelial cell proliferation. It has been characterised as a hormone secreted by immune cells, particularly macrophages, and has been linked to many inflammatory responses, including inflammation in adipose tissue [87]. Both resistin and leptin can exhibit proliferative, anti-apoptotic, pro-inflammatory effects and stimulate angiogenesis, making them potential diagnostic and prognostic biomarkers for cancer [88]. In vitro, resistin has been shown to increase levels of angiogenesis factors, including matrix metalloproteinase 2 (MMP-2) and vascular epithelial growth factor (VEGF). A positive correlation between higher levels of resistin expression and the degree of histological differentiation of EOC and the incidence of lymph node metastasis has also been shown [89]. Elevated resistin levels accompany chronic inflammation, which can induce the process of carcinogenesis in ovarian cancer [90]. Pang Li [91] conducted a study in patients with EOC (*N* = 50), showing an association of resistin expression with pathological grade (*p* = 0.017) and lymph node metastasis (*p* = 0.045) and no association with age, histotype, residual tumour after initial laparotomy, serum CA125 levels and FIGO grade [91]. 

#### 4.2.3. Adiponectin

Adiponectin is responsible for glycaemia and lipidaemia homeostasis and influences appetite control. It exhibits potent vasoprotective effects through direct action on the vascular endothelium. Adiponectin leads to vasodilation, inhibition of adhesion molecule expression, inhibition of inflammatory cytokine (TNFα) inflammation, increased nitric oxide (NO) production, stimulation of angiogenesis, and inhibition of endothelial and smooth muscle cell proliferation and migration [92]. In vitro studies by Ouh YT et al. [93] on the role of adiponectin in angiogenesis demonstrate that it stimulates CXC chemokine ligand 1 (CXCL1) secretion from ovarian cancer cells, which in turn induces angiogenesis independently of VEGF. It is suggested that this protein is a key molecule in the initiation of angiogenesis in ovarian cancer cells and may be a novel therapeutic target for the treatment of ovarian cancer [94]. Beyazit F et al. [95] showed reduced adiponectin levels in women with polycystic ovary syndrome [95]. Jin JH et al. [96] found that the mean concentrations of both adiponectin and leptin in ovarian cancer patients were lower than in the control group (8.25 vs. 11.44 μg/mL; *p* = 0.026, respectively) (7.09 vs. 15.4 ng/mL; *p* = 0.001). At the same time, they found no significant difference in adiponectin and leptin levels between early (I/II) and advanced (III/IV) disease stages (*p* = 0.078) [96]. Slomian et al. [97], investigating the role of adiponectin and leptin in ovarian cancer, found no correlation between disease stage and response to treatment and the levels of the adipokines studied. However, they noted that the value of the leptin/adiponectin (L/A) ratio can be considered as a predictor of clinical response to the applied treatment (the lower the L/A value before treatment, the better the response to the applied chemotherapy) [97]. Feng Y et al. [98] found significantly higher levels of adiponectin in serum and ascites fluid in ovarian cancer patients than in healthy patients (*p* < 0.05). Serum adiponectin expression in FIGO stage IV patients was significantly higher than in earlier stages (*p* < 0.05), with no significant difference between the other stages. In contrast, adiponectin expression in ascites at stages III and IV was significantly higher than the corresponding expression at the other stages [98]. 

#### 4.2.4. Visfatin

Visfatin (Visfatin/NAMPT, nicotinamide phosphoribosyltransferase) is a protein produced by visceral adipose tissue. Visfatin exists in two forms, extracellular (eNAMPT) and intracellular (iNAMPT). Intracellularly, it plays a regulatory role in NAD+ biosynthesis; extracellularly, visfatin is associated with many hormone-like signalling pathways and activates some intracellular signalling cascades [99]. It shares some similarities in action with insulin. It is one of the components in the link between adipose tissue and the inflammatory response, but the mechanism has not been fully explored [92]. Research by Gogola-Mruk J. et al. [100] demonstrates that visfatin acts as an anti-apoptotic factor by regulating mitochondrial activity, leading to resistance to anoikis in ovarian cancer spheroids. These findings suggest that visfatin is a potential new therapeutic target for the treatment of ovarian cancer with peritoneal dissemination [100]. Results from Nacarelli T. et al. [101] suggest that NAMPT inhibitors (such as FK866) may be repurposed to suppress therapy-induced senescence-associated CSCs for improving platinum-based standard of care, a major obstacle in the clinical management of EOC [101].

#### 4.2.5. Chemerin

Chemerin (CHEM) is a protein with a dual role in the body. As an adipokine, it is mainly synthesised by adipose tissue. It exerts paracrine and autocrine effects on adipocytes, stimulating their maturation and differentiation process. Chemerin also affects lipid and carbohydrate metabolism. Synthesised in the liver, and in smaller amounts, also in the lymph nodes, ovaries and pancreas, it acts as a chemoattractant for immune cells, regulating the innate and acquired inflammatory response [102]. Chemerin is also involved in angiogenesis and blood pressure regulation [103]. The results of Gao Ch. et al. [104] suggest that chemerin increases PD-L1 (programmed death ligand 1) expression in ovarian cancer cells and also promotes tumour cell proliferation and migration. However, the mechanism by which chemerin regulates PD-L1 requires further investigation [104]. Guzel EC. et al. [105] investigated the association of overweight/obesity with omentin and chemerin in women with PCOS. They found that body-fat mass appears to be the main determinant of increased chemerin and decreased omentin levels in women with PCOS [105]. Schmitt M. et al. [106] studied the effect of chemerin on ovarian cancer cell lines in vitro. The addition of chemerin significantly reduced the number of OVCAR-3 cells and halved the colony growth of these cells. Chemerin increased IFNα levels approximately fourfold in the culture medium of the cell lines tested. The antitumour effect of chemerin on ovarian cancer cells in vitro was mediated by activation of IFNα response genes, resulting from a chemerin-triggered increase in levels of the secreted cytokine [106].

#### 4.2.6. Apelin

Apelin is a protein released by adipocytes following insulin stimulation. Apelin is expressed in adipose tissue, CNS, heart muscle, lung and mammary gland. Apelin expression has been detected in various tissues and organs, such as the stomach, brain, heart, lung, uterus and ovary. The peptide gives rise to three active forms built sequentially from 36, 17 and 13 amino acids, and their biological activity inversely correlates with the length of the peptide chain [107]. Studies in an animal model demonstrate the involvement of apelin and its receptor APJ in angiogenesis. Its expression is significantly increased during adipocyte differentiation and maturation [77]. Hoffmann M. et al. [108] examined the expression of apelin and its receptor in non-cancerous and cancerous ovarian cell lines under the influence of bisphenol A (BPA) and its derivatives. They observed that the expression level of the APJ receptor was higher in epithelial cancer cells than in granulosa cell tumours (granuloma, folliculoma); however, the opposite applies to apelin expression and secretion. Low concentrations of BPA increased apelin expression and secretion in the epithelial ovarian cancer cell line OVCAR-3. The results of this study suggest that BPA and its derivatives induce ovarian cancer cell progression by up-regulating apelin, which acts as a mitogenic factor in these cells [108]. Neelakantan D. et al. [109] showed that apelin receptor (APJ) is a viable target that promotes tumour progression of HGSOC. In addition, they showed that increased APJ expression significantly correlates with decreased median overall survival [109].

#### 4.2.7. Omentin

Omentin (Intellectin, INTL) is mainly synthesised in visceral adipose tissue. It occurs in two isoforms: omentin-1 (INTL1), detected in the blood, and omentin-2 (INTL2), detected in the intestinal lumen. Reduced blood levels of omentin-1 are thought to be associated with insulin resistance, the development of type 2 diabetes, obesity and metabolic syndrome [110]. Omentin-1 has been shown to play a key role in cell tumourigenesis and differentiation, as well as accelerating apoptosis in cancer cells [111]. A literature review by Paval RD et al. [112] found that ITLN1 levels varied in cancer patients but were different from healthy subjects. Patients with gastrointestinal and prostate cancer showed increased levels of circulating ITLN1, whereas gynaecological and breast cancer patients had lower ITLN1 levels than controls. While analysing the studies assessing the mode of action of ITLN1, it was observed that ITLN1 can activate the PI3k/Akt pathway. Abnormal regulation of this pathway may lead to mesenchymal cell proliferation and promote cancer progression and development [112]. The studies evaluating the association between ITLN1 and gynaecological cancers have yielded inconclusive results. In a meta-analysis by Arjmand et al. [113], circulating ITLN1 was not significantly different between women with ovarian cancer and healthy controls [113]. In contrast, Au-Yeung C.L. et al. [114] showed that circulating ITLN1 was lower in patients with high-grade ovarian cancer compared with a group of healthy women and women with benign gynaecological disease. They also showed that ITLN1 mRNA expression was lower in the omental adipose tissue of patients with high-grade ovarian cancer compared to women with benign disease. In addition, their study showed that the pro-inflammatory cytokines TNF-α and TGF-β in the omental microenvironment downregulate ITLN1 expression in mesothelial cells in HGSC patients, and also that recombinant ITLN1 inhibits OC growth in vivo. The researchers also detected a significant inverse correlation between CA125 and ITLN1 levels (r = −0.394; *p* < 0.001). They found that CA125 had a significantly greater area under the curve (AUC) than omentin (*p* = 0.0031), and CA125 in combination with ITLN1 had a significantly greater AUC than CA125 alone (*p* = 0.0295) or ITLN1 alone (*p* = 5.095 × 10^−6^), suggesting that ITLN1 complemented CA125 in identifying OC patients [114]. Summarised information on the role of adipocytokines in ovarian cancer is presented in Table 3. 

### 4.3. Cancer Stem Cells (CSCs)

Cancer stem cells (CSCs) are a small subpopulation of tumour cells (0.01% to 0.1%) [115] which exist within ovarian cancer and have the ability to self-renew [116]. Ovarian CSCs are thought to be responsible for tumour growth, metastasis and recurrence and also resistance to chemotherapy [116,117]. The information on selected cancer stem cell markers is summarised in Table 4.

### 4.4. microRNAs (miRNAs)

The role of miRNA regulation in ovarian cancer pathology is still largely unknown. Studies on this topic account for less than 4% of all published reports. Understanding the mechanisms by which abnormal expression of miRNAs and their target genes affect ovarian cancer initiation, proliferation and resistance to chemotherapy may contribute to the development of new therapeutic strategies for the treatment of these cancers [107]. MicroRNAs are short, non-coding single-stranded molecules of 19–25 nucleotides in length, involved in regulating the expression of many genes [143,144]. MicroRNAs play a key role in normal physiological functions, and altered expression of specific miRNAs has been linked to many diseases [145]. These molecules control biological processes such as proliferation, cell differentiation, angiogenesis, migration, apoptosis or oncogenesis. The involvement of miRNAs in the development of cancer has been demonstrated. Detection of individual miRNAs and monitoring of changes in their expression profile may be useful in the early detection of cancer cells. They may also serve as a prognostic factor for the course of the disease as well as its treatment [146]. Some of the miRNAs previously identified in cells and tissues have also been found in extracellular fluids such as plasma, serum, saliva and urine [147]. MicroRNAs can also be detected in breast milk, tears, colostrum, bronchoalveolar lavage, semen and pleural fluid [93]. The discovery that the miRNA profile in plasma often mirrors miRNAs in tumour tissue makes these molecules a readily available marker [148]. Summaries of miRNA molecules with increased and decreased expression in ovarian cancer are shown in Table 5 and Table 6, respectively.

Teng et al. [154] conducted a meta-analysis of five clinical trials to determine the diagnostic value of miR-200a-3p and miR-200c-3p molecules in patients with EOC. The studies suggest that miR-200a/c may contribute to the progression of EOC by affecting the cellular adhesion process. A strong association between miR-200a/c and overall survival in patients with EOC has also been demonstrated [154]. Zhu Z et al. [155] showed that miR-205 levels were elevated in ovarian cancer patients. A combined panel of miR-205, CA125 and HE4 performed best in detecting early-stage ovarian cancer [155]. In a study by Ali FT. at all [5], it was indicated that microRNA-204 could serve as a useful biomarker for the detection of ovarian cancer. This is the first study to show that the combination of microRNA-204, CA125 and CA19.9 is the best test for the early detection of ovarian tumours. The combination of microRNA-204, CA125 and CA19.9 showed the highest diagnostic performance with an AUC of 1000 (*p* < 0.001) [5]. A comparison of the sensitivity and specificity of selected miRNA molecules is shown in Table 7.

Vang S. et al. [156] compared the expression profile of miRNA molecules in primary serous ovarian cancer and in metastatic lesions. They demonstrated, among other things, the important role of miR-146a and miR-150 molecules in metastasis and showed their association with cisplatin resistance [156,157]. An important regulatory mechanism in the development and progression of ovarian cancer is the activity of miRNA molecules. The researchers’ attention is drawn to the elevated expression of miR-199a, miR-200a and miR-214 and the decreased expression of miR-100 and miR-214, targeting the tumour suppressor gene PTEN and associated with platinum resistance [157]. Zhou et al. [93] showed that urinary miR30a-5p expression was significantly elevated in patients with serous ovarian adenocarcinoma (*N* = 39) compared with expression in benign gynaecological disease (*N* = 26) and healthy controls (*N* = 30). This study also showed a significant reduction in miR-30a-5p expression in the urine of patients who underwent tumour resection, which suggests an origin of this molecule from ovarian serous adenocarcinoma itself, making it a diagnostic and therapeutic marker [93]. Given the resistance of miRNAs to degradation by ribonucleases and their availability in plasma exosomes, these molecules may serve as novel biomarkers for cancer detection, therapeutic evaluation and prognosis [158].

### 4.5. Autoantibodies (AABs)

Tumour cells can induce an immune response resulting in the production of autoantibodies associated with the resulting tumour. These autoantibodies have been detected in serum for many tumours at an early stage of tumour development, before the onset of clinical symptoms [159]. Autoantibodies against tumour-associated proteins can identify tumours that are too small to secrete a sufficiently large amount of protein biomarkers for detection, and therefore they are more rapidly detected in serum than, for example, the biomarker CA125. The use of proteomics makes it possible not only to identify new protein biomarkers, but also to find proteins related to human immunoglobulins and to detect autoantibodies [15]. Recent studies indicate that anti-TP53 autoantibodies may serve as a biomarker for ovarian cancer. A study by Yang W-L, et al. [160] suggests that only 20–25% of patients with invasive epithelial ovarian cancer had elevated levels of anti-TP53 autoantibodies. They concluded that this biomarker may be limited, though potentially important, in detecting early-stage disease. They also showed that TP53 autoantibody levels could be complementary with CA125 determination and could be part of a panel of biomarkers that would significantly improve the efficacy of CA125. According to the researchers, anti-TP53 autoantibody titres can grow for 8–12 months before CA125 determination [15,160]. Currently, research projects are focused on the use of autoantibody combinations for the diagnosis of early-stage ovarian cancer. A comparison of the sensitivity and specificity of selected autoantibody combinations is shown in Table 8.

### 4.6. Circulating DNA (ctDNA)

ctDNA are DNA fragments that are released from tumour tissues into blood, urine and other body fluids through apoptosis, necrosis, lysis and active secretion [22]. An analysis of ctDNA, the so-called ‘liquid biopsy’, provides a potential non-invasive method for tumour detection and monitoring [164]. ctDNA can be detected and quantified with PCR, BEAMing technology and sequencing. Tumour tissues are characterised by specific genetic alterations such as point mutations, copy number changes, deletions and epigenetic changes. Studies have shown that these cancer-related alterations are also present in ctDNA, even in patients in the early stages of the disease [22]. Lu Y et al. [165] conducted a meta-analysis of eight studies evaluating the association between ctDNA and prognosis in patients with epithelial ovarian cancer. Their results suggest that women with high ctDNA levels have a poor prognosis [165].

## 5. Summary

In spite of the numerous studies carried out, the search is still ongoing for new markers and combinations of markers, the use of which would significantly improve the detection of ovarian cancer and thus contribute to a reduction in female mortality. The are grounds for hope with regard to the potential use of selected immunological and molecular parameters in combination with previously used markers for the diagnosis of ovarian cancer, which is currently the subject of numerous studies aimed at their potential use in the development of new diagnostic and therapeutic strategies in patients with ovarian cancer.

## Figures and Tables

**Table 1:** Summary of features of Type I and Type II ovarian cancer [2,3,4,5,6,7].

	Type I	Type II
**subtypes**	serous carcinoma G1 and G2 endometrioid carcinoma G1 and G2 mucinous carcinoma clear cell carcinoma	serous carcinoma G3 endometrioid non-squamous carcinoma G3 germ carcinoma G3 sarcoma
**genetic stability**	stable	unstable
**mutations**	KRAS, PTEN, BRAF, PAX8	TP53
**BRCA germline gene mutations**	rare	frequent
**recognised**	in early stages	in FIGO stage III and IV
**growth**	free	quick
**sensitivity to chemotherapy**	small	large
**relapses**	rarer	quick
**median concentration CA125**	53–413 U/mL	395–1340 U/mL
**5-year survival period**	95%	<30%
**prognosis**	10% of deaths	90% of deaths

KRAS—kirsten rat sarcoma viral oncogene homolog, PTEN—phosphatase, suppressor protein (phosphatase and tensin homolog), BRAF—proto-oncogene (B-Raf proto-oncogene), PAX8—transcription factor (Paired box gene 8); FIGO—International Federation of Gynecology and Obstetrics (Fédération Internationale de Gynécologie et d’Obstétrique).

**Table 2:** Role of selected galectins in ovarian cancer [65,66,68,69,70,71,73,74,75,76].

Galectin	Expression in Tissue	Serum Concentration
**GAL-1**	increased in primary ovarian cancer tissue; positive correlation with tumour stage and lower susceptibility to chemotherapy with cisplatin [65]	significant association with FIGO stage (III, IV > I, II) [68] elevated in epithelial ovarian carcinomas; higher in serous subtype [69]
**GAL-3**	higher in clear cell carcinoma and serous and mucinous tumours than in endometrial cancer and transitional tumours; lack of differentiation between benign, borderline and transitional cancers [75]	a significant correlation between high serum levels and the presence of metastases; higher values in patients with recurrent ovarian cancer compared to women with stable disease [76]
	increased expression in the EOC in the absence of expression in normal ovarian tissues; association of increased expression with shorter progression-free survival (PFS) [70]	
	reduced expression in cyst fluid and serum of patients with early-stage disease [71]	
**GAL-7**	increased expression in benign and malignant tumours compared to healthy tissue; association of increased expression with tumour progression through increased invasiveness of cancer cells and proapoptotic effects on immune cells [66,72]	
**GAL-8**	association of expression with prolonged prognostic period for overall and disease-free survival of patients [74]	increased plasma levels in patients with serous carcinoma; association of high plasma concentrations with shorter OS and DFS [73]
**GAL-9**	correlation of moderate expression with shortened survival; high Gal-9 expression associated with better prognosis [74]	association of high plasma concentrations with shorter OS and DFS [73]

**Table 3:** Role of selected adipocytokines in ovarian cancer [80,82,84,86,89,91,96,97,99,100,101,106,108,109,112,113,114].

Adipocytokine	Role
Leptin	high co-expression of LEP and LEPR correlating with poor survival of ovarian cancer patients; leptin/LEPR signalling via JAK2/STAT3 has the potential to significantly impact pathogenesis in a subset of ovarian cancer patients [84]
	stimulation of proliferation of ovarian cancer cell lines in vitro [80]
	association of overexpression of leptin and its receptor in tissue with aggressive nature of the disease [86]
	stimulation of proliferation and inhibition of apoptosis through activation of PI3K/AKT signalling pathway [82]
Resistin	positive correlation between expression levels or the degree of EOC differentiation and lymph node metastasis [89,91]
	association of serum levels with chronic inflammation, leading to the induction of ovarian carcinogenesis [80]
Adiponectin	concentration lower in ovarian cancer patients than in controls; no association with stage of disease progression [96]
	no association of concentration with stage of disease and response to treatment [97]
	concentration higher in ovarian cancer patients than in controls; relationship of expression to FIGO stage [99]
Visfatin	anti-apoptotic factor leading to anoikis resistance in ovarian cancer spheroids [100]
	NAMPT inhibition suppresses senescence-associated CSCs induced by platinum-based chemotherapy in EOC [101]
Chemerin	promotion of proliferation and migration of ovarian cancer cells by upregulating expression of PD-L1 [104]
	activation of IFNα response by chemerin; increased production of IFNα protein in ovarian cancer cells and reduction in cancer cell numbers in vitro [106]
Apelin	BPA and its derivatives induce ovarian cancer cell progression by up-regulating apelin, which acts as a mitogenic factor in these cells [108]
	increased APJ expression significantly correlates with decreased median overall survival [109]
Omentin	activation of the PI3k/Akt pathway [112]
	no relationship between ITNL1 levels in patients with ovarian cancer and healthy control [113]
	relationship between ITLN1 in patients with high-grade ovarian cancer, healthy women and women with benign gynaecologic disease [114]

**Table 4:** Selected markers of cancer stem cells [118,119,120,121,122,123,124,125,126,127,128,129,130,131,132,133,134,135,136,137,138,139,140,141,142].

Marker of CSC	Role
CA24	independent prognostic marker for survival of ovarian cancer patients; development, invasion and metastasis of tumour cells [119]
	correlation of expression with FIGO stage and the presence of peritoneal and lymph node metastases; significant association with progression-free survival and overall survival in ovarian cancer patients [120]
	tumour formation, metastasis, resistance to chemotherapy, poor prognosis, relapse [118,120,121]
CD44	tumour formation, progression, resistance to chemotherapy, poor prognosis, relapse [118,122]
	significant association of CD44 expression with high TMN (classification of malignant tumours) stage and low five-year OS (overall survival) rates [123]
	no correlation with disease-free survival (DFS) and tumour grade, lymph node metastases, age of patients, residual tumour size, ascites volume and response to chemotherapy [123]
	association of CD44s isoform overexpression with poor OS and worse DFS and resistance to chemotherapy; no association between overexpression of the CD44v6 isoform and bad OS [124]
	the association of high CD44 expression with higher histological grade and more advanced FIGO stage and with worse OS and DFS [125]
CD117	tumour formation, resistance to chemotherapy, poor prognosis [118,126]
	significant relationship of CD117 expression with FIGO stage, histological type, degree of tumour differentiation and age, worse OS; no statistically significant association between CD117 expression and DFS [127]
	CD117 expression statistically correlated with response to chemotherapy; CD117+ patients less sensitive to chemotherapy than CD117 - patients; human ovarian cancer cells with the CD117(+) phenotype have unique properties of CSCs, including self-renewal, differentiation, high tumourigenic potential and chemoresistance [128]
CD133	tumour formation, progression, chemoresistance, poor prognosis, successful treatment [129,130]
	strong correlation of CD133 expression with poor two-year overall survival (OS); correlation of CD133 expression with tumour stage;
	no association with patient age, tumour grade, histological type or response to treatment [131]
	correlation of CD133 expression with FIGO stage and with degree of tumour differentiation [124]
	no association between CD133 expression and progression-free survival (PFS) or OS in patients with serous ovarian cancer; no significant association between CD133 expression in tumour cells and age, serum CA125 levels and tumour grade [132]
	lack of CD133 expression in patients with primary epithelial ovarian cancer significantly associated with high sensitivity to platinum in patients with and without central nervous system (CNS) metastases present [133]
	association between CD133 expression in primary tumours and increased risk of CNS metastases [133]
ALDH1	cell proliferation and migration promotion, poor survival, chemoresistance [134,135,136]
	association of high ALDH1 expression in ovarian cancer cells with early FIGO stage, degree of good differentiation, better survival and favourable prognosis [137]
	significant relationship of elevated expression of ALDH1 with bad OS, but not with DFS; association of elevated ALDH1 with FIGO stage, lymph node metastasis and distant metastasis [138]
	correlation of ALDH1 overexpression with poor OS as well as with worse DFS [124]
	association of increased ALDH1 expression in post-treatment advanced epithelial ovarian cancer patients with poor response to chemotherapy [139]
SOX2	cancer stem cell maintenance and self-renewal, poor prognosis and chemoresistance [118]
	association between SOX2 expression and poor prognosis in ovarian cancer; SOX2 expression associated with reduced DFS duration, no association between SOX2 expression and OS; significant association between SOX2 expression and high-grade serous carcinoma; no significant correlation between SOX2 expression and response to chemotherapy [140]
	association between non-radical tumour removal surgery and shorter OS and PFS in patients with SOX2-positive tumours [141]; association of SOX2 overexpression in paclitaxel-resistant cells [142]

**Table 5:** miRNAs with increased expression in ovarian cancer [149,150,151,152,153].

EOC	SOC	CCC
miR-21, miR-92, miR-93, miR-126 miR-29a [149] miR-132, miR-26a, let-7b, miR-145 [152] miR-203 [153]	miR-200a, miR-200b, miR-200c, miR-20a, miR-21, miR-23a, miR-23b, miR-27a, miR-141, miR-16 [150] miR-200c-3p [151]	miR-182-5p, miR-200a-5p [151]

**Table 6:** miRNAs with reduced expression in ovarian cancer [149,150,151].

EOC	SOC	CCC
miR-155, miR-127 miR-99b [149]	miR-214, miR-26a, miR-29a, let-7b, miR-100, miR-10b, miR-125a, miR- 125b, miR-143, miR-145, miR-199a-AS, miR-99° [150]	miR-383 [151]

**Table 7:** Comparison of sensitivity and specificity of miRNA molecules according to [154,155,156].

miRNA	Sensitivity	Specificity
miR-200a-3p	84%	83%
(for EOC)		
miR-200c-3p	75%	66%
(for EOC)		
Exosomal miR-205	66.7%	78.1%
(for OC)		
Exosomal miR-205, CA125 and HE4	100%	86.1%
(for OC)		

**Table 8:** Comparison of sensitivity and specificity of selected autoantibody combinations. Based on [22,161,162,163].

Autoantibodies	Sensitivity/Specificity
anti-PT53, GNAS, NPMI [127]	51.2%/86%
against p53, c-MYC, p90, p62, alpha 2-HS glycoprotein (AHSG), 14-3-3 zeta, RAS-like proto-oncogene A (RalA), KH domain-containing protein overexpressed in cancer (Koc), and P16 [162]	61.4%/85%
AABs against survivin, p53, p16, cyclin B1, cyclin D1, cyclin A, cyclin E, Koc, IGF2 mRNA-binding protein 1 (IMP1), P62, cyclin-dependent kinase 2 (CDK2), P90, and c-MYC [163]	62.5%/85%

## Data Availability

Not applicable.

## References

[1] De Leo A., Santini D., Ceccarelli C., Santandrea G., Palicelli A., Acquaviva G., Chiarucci F., Rosini F., Ravegnini G., Pession A. (2021). What Is New on Ovarian Carcinoma: Integrated Morphologic and Molecular Analysis Following the New 2020 World Health Organization Classification of Female Genital Tumors. Diagnostics.

[2] Shih I.e.M., Kurman R.J. (2004). Ovarian tumorigenesis: A proposed model based on morphological and molecular genetic analysis. Am. J. Pathol..

[3] Kurman R.J., Shih I.M. (2008). Pathogenesis of ovarian cancer: Lessons from morphology and molecular biology and their clinical implications. Int. J. Gynecol. Pathol..

[4] Charkhchi P., Cybulski C., Gronwald J., Wong F.O., Narod S.A., Akbari M.R. (2020). CA125 and Ovarian Cancer: A Comprehensive Review. Cancers.

[5] Ali F.T., Soliman R.M., Hassan N.S., Ibrahim A.M., El-Gizawy M.M., Mandoh A.A.Y., Ibrahim E.A. (2022). Sensitivity and specificity of microRNA-204, CA125, and CA19.9 as biomarkers for diagnosis of ovarian cancer. PLoS ONE.

[6] Atallah G.A., Abd Aziz N.H., Teik C.K., Shafiee M.N., Kampan N.C. (2021). New Predictive Biomarkers for Ovarian Cancer. Diagnostics.

[7] Nowak-Markwitz E., Spaczyński M. (2012). Ovarian cancer—Modern approach to its origin and histogenesis. Ginekol. Pol..

[8] Białas P., Jankowska A. (2015). Biochemical markers in breast and ovarian cancer. Pol. Rev. Health Sci..

[9] Bast R.C., Feeney M., Lazarus H., Nadler L.M., Colvin R.B., Knapp R.C. (1981). Reactivity of a monoclonal antibody with human ovarian carcinoma. J. Clin. Investig..

[10] Bast R.C., Klug T.L., St John E., Jenison E., Niloff J.M., Lazarus H., Berkowitz R.S., Leavitt T., Griffiths C.T., Parker L. (1983). A radioimmunoassay using a monoclonal antibody to monitor the course of epithelial ovarian cancer. N. Engl. J. Med..

[11] Ueda Y., Enomoto T., Kimura T., Miyatake T., Yoshino K., Fujita M., Kimura T. (2010). Serum biomarkers for early detection of gynecologic cancers. Cancers.

[12] Liu S., Wu M., Wang F. (2021). Research Progress in Prognostic Factors and Biomarkers of Ovarian Cancer. J. Cancer.

[13] Chen M., Lei N., Tian W., Li Y., Chang L. (2022). Recent advances of non-coding RNAs in ovarian cancer prognosis and therapeutics. Ther. Adv. Med. Oncol..

[14] Elias K.M., Guo J., Bast R.C. (2018). Early Detection of Ovarian Cancer. Hematol. Clin. North. Am..

[15] Yang W.L., Lu Z., Bast R.C. (2017). The role of biomarkers in the management of epithelial ovarian cancer. Expert Rev. Mol. Diagn..

[16] Yurkovetsky Z., Skates S., Lomakin A., Nolen B., Pulsipher T., Modugno F., Marks J., Godwin A., Gorelik E., Jacobs I. (2010). Development of a multimarker assay for early detection of ovarian cancer. J. Clin. Oncol..

[17] Van Calster B., Valentin L., Van Holsbeke C., Zhang J., Jurkovic D., Lissoni A.A., Testa A.C., Czekierdowski A., Fischerová D., Domali E. (2011). A novel approach to predict the likelihood of specific ovarian tumor pathology based on serum CA-125: A multicenter observational study. Cancer Epidemiol. Biomark. Prev..

[18] Al Musalhi K., Al Kindi M., Al Aisary F., Ramadhan F., Al Rawahi T., Al Hatali K., Mula-Abed W.A. (2016). Evaluation of HE4, CA-125, Risk of Ovarian Malignancy Algorithm (ROMA) and Risk of Malignancy Index (RMI) in the Preoperative Assessment of Patients with Adnexal Mass. Oman Med. J..

[19] Lee M., Chang M.Y., Yoo H., Lee K.E., Chay D.B., Cho H., Kim S., Kim Y.T., Kim J.H. (2016). Clinical Significance of CA125 Level after the First Cycle of Chemotherapy on Survival of Patients with Advanced Ovarian Cancer. Yonsei Med. J..

[20] Kirchhoff C., Habben I., Ivell R., Krull N. (1991). A major human epididymis-specific cDNA encodes a protein with sequence homology to extracellular proteinase inhibitors. Biol. Reprod..

[21] Rowswell-Turner R.B., Singh R.K., Urh A., Yano N., Kim K.K., Khazan N., Pandita R., Sivagnanalingam U., Hovanesian V., James N.E. (2021). HE4 Overexpression by Ovarian Cancer Promotes a Suppressive Tumor Immune Microenvironment and Enhanced Tumor and Macrophage PD-L1 Expression. J. Immunol..

[22] Zhang R., Siu M.K.Y., Ngan H.Y.S., Chan K.K.L. (2022). Molecular Biomarkers for the Early Detection of Ovarian Cancer. Int. J. Mol. Sci..

[23] Bingle L., Singleton V., Bingle C.D. (2002). The putative ovarian tumour marker gene HE4 (WFDC2), is expressed in normal tissues and undergoes complex alternative splicing to yield multiple protein isoforms. Oncogene.

[24] Galgano M.T., Hampton G.M., Frierson H.F. (2006). Comprehensive analysis of HE4 expression in normal and malignant human tissues. Mod. Pathol..

[25] Li J., Dowdy S., Tipton T., Podratz K., Lu W.G., Xie X., Jiang S.W. (2009). HE4 as a biomarker for ovarian and endometrial cancer management. Expert Rev. Mol. Diagn..

[26] Scaletta G., Plotti F., Luvero D., Capriglione S., Montera R., Miranda A., Lopez S., Terranova C., De Cicco Nardone C., Angioli R. (2017). The role of novel biomarker HE4 in the diagnosis, prognosis and follow-up of ovarian cancer: A systematic review. Expert Rev. Anticancer. Ther..

[27] Moore R.G., Hill E.K., Horan T., Yano N., Kim K., MacLaughlan S., Lambert-Messerlian G., Tseng Y.D., Padbury J.F., Miller M.C. (2014). HE4 (WFDC2) gene overexpression promotes ovarian tumor growth. Sci. Rep..

[28] Piovano E., Attamante L., Macchi C., Cavallero C., Romagnolo C., Maggino T., Landoni F., Gadducci A., Sartori E., Gion M. (2014). The role of HE4 in ovarian cancer follow-up: A review. Int. J. Gynecol. Cancer.

[29] Janas Ł., Głowacka E., Wilczyński J.R., Malinowski A., Nowak M. (2015). Evaluation of applicability of HE4 and ROMA in the preoperative diagnosis of adnexal masses. Ginekol. Pol..

[30] Moore R.G., McMeekin D.S., Brown A.K., DiSilvestro P., Miller M.C., Allard W.J., Gajewski W., Kurman R., Bast R.C., Skates S.J. (2009). A novel multiple marker bioassay utilizing HE4 and CA125 for the prediction of ovarian cancer in patients with a pelvic mass. Gynecol. Oncol..

[31] Han L.N., Han Y.W., Yan P. (2022). Prognostic values of human epididymis protein 4 expression in patients with endometrial cancer: A systematic review and meta-analysis. J. Obstet. Gynaecol. Res..

[32] Ghafoor A., Thomas A., Hassan R. (2018). Targeting mesothelin in ovarian cancer. Oncotarget.

[33] Barczyński B., Frąszczak K., Polak G., Kotarski J. (2013). Mesothelin: A novel biomarker of ovarian cancer?. GinPolMedProject.

[34] Hilliard T.S. (2018). The Impact of Mesothelin in the Ovarian Cancer Tumor Microenvironment. Cancers.

[35] McIntosh M.W., Drescher C., Karlan B., Scholler N., Urban N., Hellstrom K.E., Hellstrom I. (2004). Combining CA 125 and SMR serum markers for diagnosis and early detection of ovarian carcinoma. Gynecol. Oncol..

[36] Badgwell D., Lu Z., Cole L., Fritsche H., Atkinson E.N., Somers E., Allard J., Moore R.G., Lu K.H., Bast R.C. (2007). Urinary mesothelin provides greater sensitivity for early stage ovarian cancer than serum mesothelin, urinary hCG free beta subunit and urinary hCG beta core fragment. Gynecol. Oncol..

[37] Giordano G., Ferioli E., Tafuni A. (2022). The Role of Mesothelin Expression in Serous Ovarian Carcinoma: Impacts on Diagnosis, Prognosis, and Therapeutic Targets. Cancers.

[38] Li Y., Tian W., Zhang H., Zhang Z., Zhao Q., Chang L., Lei N., Zhang W. (2022). MSLN Correlates With Immune Infiltration and Chemoresistance as a Prognostic Biomarker in Ovarian Cancer. Front. Oncol..

[39] Rittling S.R. (2011). Osteopontin in macrophage function. Expert Rev. Mol. Med..

[40] Lan Z., Fu D., Yu X., Xi M. (2016). Diagnostic values of osteopontin combined with CA125 for ovarian cancer: A meta-analysis. Fam. Cancer.

[41] Wang Y.D., Chen H., Liu H.Q., Hao M. (2014). Correlation between ovarian neoplasm and serum levels of osteopontin: A meta-analysis. Tumour. Biol..

[42] Ye B., Skates S., Mok S.C., Horick N.K., Rosenberg H.F., Vitonis A., Edwards D., Sluss P., Han W.K., Berkowitz R.S. (2006). Proteomic-based discovery and characterization of glycosylated eosinophil-derived neurotoxin and COOH-terminal osteopontin fragments for ovarian cancer in urine. Clin. Cancer Res..

[43] Anastasi E., Manganaro L., Granato T., Benedetti Panici P., Frati L., Porpora M.G. (2013). Is CA72-4 a useful biomarker in differential diagnosis between ovarian endometrioma and epithelial ovarian cancer?. Dis. Markers.

[44] Wang Q., Wu Y., Zhang H., Yang K., Tong Y., Chen L., Zhou Q., Guan S. (2019). Clinical Value of Serum HE4, CA125, CA72-4, and ROMA Index for Diagnosis of Ovarian Cancer and Prediction of Postoperative Recurrence. Clin. Lab..

[45] Li M., Men X., Zhang X. (2020). Diagnostic value of carbohydrate antigen 72-4 combined with carbohydrate antigen 15.3 in ovarian cancer, cervical cancer and endometrial cancer. J. BUON.

[46] Liu L., Liu J., Dai S., Wang X., Wu S., Wang J., Huang L., Xiao X., He D. (2007). Reduced transthyretin expression in sera of lung cancer. Cancer Sci..

[47] Zheng X., Chen S., Li L., Liu X., Liu X., Dai S., Zhang P., Lu H., Lin Z., Yu Y. (2018). Evaluation of HE4 and TTR for diagnosis of ovarian cancer: Comparison with CA-125. J. Gynecol. Obstet. Hum. Reprod..

[48] Kozak K.R., Su F., Whitelegge J.P., Faull K., Reddy S., Farias-Eisner R. (2005). Characterization of serum biomarkers for detection of early stage ovarian cancer. Proteomics.

[49] Kim Y.W., Bae S.M., Lim H., Kim Y.J., Ahn W.S. (2012). Development of multiplexed bead-based immunoassays for the detection of early stage ovarian cancer using a combination of serum biomarkers. PLoS ONE.

[50] Denel M., Marczak A. (2013). Panels of protein biomarkers and non-protein markers in the diagnosis of the ovarian cancer. Przegląd Menopauzalny.

[51] Kim B., Park Y., Kim B., Ahn H.J., Lee K.A., Chung J.E., Han S.W. (2019). Diagnostic performance of CA 125, HE4, and risk of Ovarian Malignancy Algorithm for ovarian cancer. J. Clin. Lab. Anal..

[52] Barr C.E., Funston G., Jeevan D., Sundar S., Mounce L.T.A., Crosbie E.J. (2022). The Performance of HE4 Alone and in Combination with CA125 for the Detection of Ovarian Cancer in an Enriched Primary Care Population. Cancers.

[53] Dochez V., Caillon H., Vaucel E., Dimet J., Winer N., Ducarme G. (2019). Biomarkers and algorithms for diagnosis of ovarian cancer: CA125, HE4, RMI and ROMA, a review. J. Ovarian Res..

[54] Basta A., Bidziński M., Bieńkiewicz A., Blecharz P., Bodnar L., Jach R., Knapp P., Kojs Z., Kotarski J., Markowska J. (2017). Recommendations of the Polish Gynecological Oncology Society for the diagnosis and treatment of ovarian cancer. Curr. Gynecol. Oncol..

[55] Braicu E.I., Krause C.L., Torsten U., Mecke H., Richter R., Hellmeyer L., Lanowska M., Müller B., Koch E., Boenneß-Zaloum J. (2022). HE4 as a serum biomarker for the diagnosis of pelvic masses: A prospective, multicenter study in 965 patients. BMC Cancer.

[56] Shin K.H., Kim H.H., Kwon B.S., Suh D.S., Joo J.K., Kim K.H. (2020). Clinical Usefulness of Cancer Antigen (CA) 125, Human Epididymis 4, and CA72-4 Levels and Risk of Ovarian Malignancy Algorithm Values for Diagnosing Ovarian Tumors in Korean Patients With and Without Endometriosis. Ann. Lab. Med..

[57] Jacobs I., Oram D., Fairbanks J., Turner J., Frost C., Grudzinskas J.G. (1990). A risk of malignancy index incorporating CA 125, ultrasound and menopausal status for the accurate preoperative diagnosis of ovarian cancer. Br. J. Obstet. Gynaecol..

[58] Al-Asadi J.N., Al-Maliki S.K., Al-Dahhhan F., Al-Naama L., Suood F. (2018). The accuracy of risk malignancy index in prediction of malignancy in women with adnexal mass in Basrah, Iraq. Niger. J. Clin. Pract..

[59] Kumari S. (2018). Serum Biomarker Based Algorithms in Diagnosis of Ovarian Cancer: A Review. Indian J. Clin. Biochem..

[60] Dunton C.J., Hutchcraft M.L., Bullock R.G., Northrop L.E., Ueland F.R. (2021). Salvaging Detection of Early-Stage Ovarian Malignancies When CA125 Is Not Informative. Diagnostics.

[61] Shimada C., Xu R., Al-Alem L., Stasenko M., Spriggs D.R., Rueda B.R. (2020). Galectins and Ovarian Cancer. Cancers.

[62] Chetry M., Thapa S., Hu X., Song Y., Zhang J., Zhu H., Zhu X. (2018). The Role of Galectins in Tumor Progression, Treatment and Prognosis of Gynecological Cancers. J. Cancer.

[63] Mohamed R.M., Emam A., Abdelfattah M.M., Abdel-Mageed A.I., Abdelhafeez M.A., Helwa R. (2022). Assessment of galectins -1, -3, -4, -8, and -9 expression in ovarian carcinoma patients with clinical implications. World J. Surg. Oncol..

[64] Kim H.J., Jeon H.K., Cho Y.J., Park Y.A., Choi J.J., Do I.G., Song S.Y., Lee Y.Y., Choi C.H., Kim T.J. (2012). High galectin-1 expression correlates with poor prognosis and is involved in epithelial ovarian cancer proliferation and invasion. Eur. J. Cancer.

[65] Zhang P., Shi B., Zhou M., Jiang H., Zhang H., Pan X., Gao H., Sun H., Li Z. (2014). Galectin-1 overexpression promotes progression and chemoresistance to cisplatin in epithelial ovarian cancer. Cell Death Dis..

[66] Bieg D., Bednarek I. (2016). Galectins—Abnormal expression and role in ovarian, breast and cervical carcinogenesis. Post. Biol. Komórki..

[67] Goud N.S., Bhattacharya A. (2021). Human Galectin-1 in Multiple Cancers: A Privileged Molecular Target in Oncology. Mini. Rev. Med. Chem..

[68] Abdelwahab M., Ebian H., Ibrahim T., Badr M., Lashin M., Yassin M., Ismail A., Obaya A. (2019). Clinical significance of serum galectin-1 and its tissue immunohistochemical expression in serous ovarian carcinoma patients. J. Obstet. Gynecol..

[69] Masoodi M., Shah Z.A., Beigh A.H., Ahmad S.Z., Mir A.W., Yasin B., Rasool R., Masoodi K.Z., Bhat G.M. (2021). Galectin-1 as a predictive biomarker in ovarian cancer. J. Ovarian Res..

[70] Kim M.K., Sung C.O., Do I.G., Jeon H.K., Song T.J., Park H.S., Lee Y.Y., Kim B.G., Lee J.W., Bae D.S. (2011). Overexpression of Galectin-3 and its clinical significance in ovarian carcinoma. Int. J. Clin. Oncol..

[71] Cortesi L., Rossi E., Della Casa L., Barchetti A., Nicoli A., Piana S., Abrate M., La Sala G.B., Federico M., Iannone A. (2011). Protein expression patterns associated with advanced stage ovarian cancer. Electrophoresis.

[72] Labrie M., Vladoiu M.C., Grosset A.A., Gaboury L., St-Pierre Y. (2014). Expression and functions of galectin-7 in ovarian cancer. Oncotarget.

[73] Labrie M., De Araujo L.O.F., Communal L., Mes-Masson A.M., St-Pierre Y. (2017). Tissue and plasma levels of galectins in patients with high grade serous ovarian carcinoma as new predictive biomarkers. Sci. Rep..

[74] Schulz H., Kuhn C., Hofmann S., Mayr D., Mahner S., Jeschke U., Schmoeckel E. (2018). Overall Survival of Ovarian Cancer Patients Is Determined by Expression of Galectins-8 and -9. Int. J. Mol. Sci..

[75] Lee J.H., Zhang X., Shin B.K., Lee E.S., Kim I. (2009). Mac-2 binding protein and galectin-3 expression in mucinous tumors of the ovary: An annealing control primer system and immunohistochemical study. Pathology.

[76] Anastasi E., Gigli S., Santulli M., Tartaglione S., Ballesio L., Porpora M.G., Granato T., Catalano C., Angeloni A., Manganaro L. (2017). Role of Galectin-3 Combined with Multi- Detector Contrast Enhanced Computed Tomography in Predicting Disease Recurrence in Patients with Ovarian Cancer. Asian Pac. J. Cancer Prev..

[77] Jasińska A., Pietruczuk M. (2010). Adipocytokines—Proteins of multidirectional function. J. Lab. Diagn..

[78] Trayhurn P. (2022). Adipokines: Inflammation and the pleiotropic role of white adipose tissue. Br. J. Nutr..

[79] Murawska-Ciałowicz E. (2017). Adipose tissue—Morphological and biochemical characteristic of different depots. Postep. Hig. Med. Dosw..

[80] Kwiatkowska K., Pawłowska A., Suszczyk D., Bilska M., Tarkowski R., Kotarski J., Wertel I. (2017). Does obesity increase the risk of ovarian cancer? A literature review. Curr. Gynecol. Oncol..

[81] Gogga P., Karbowska J., Meissner W., Kochan Z. (2011). Role of leptin in the regulation of lipid and carbohydrate metabolism. Postep. Hig. Med. Dosw..

[82] Uddin S., Bu R., Ahmed M., Abubaker J., Al-Dayel F., Bavi P., Al-Kuraya K.S. (2009). Overexpression of leptin receptor predicts an unfavorable outcome in Middle Eastern ovarian cancer. Mol. Cancer.

[83] Gu F., Zhang H., Yao L., Jiang S., Lu H., Xing X., Zhang C., Jiang P., Zhang R. (2019). Leptin contributes to the taxol chemoresistance in epithelial ovarian cancer. Oncol. Lett..

[84] Kumar J., Fang H., McCulloch D.R., Crowley T., Ward A.C. (2017). Leptin receptor signaling via Janus kinase 2/Signal transducer and activator of transcription 3 impacts on ovarian cancer cell phenotypes. Oncotarget.

[85] Kim K.T., Kim Y.J., Moon H. (2006). Expression of immunoreactive leptin and its receptor on tchem cell growth and proliferation in the ovarian cancer: A preliminary report. Int. J. Gynecol. Cancer.

[86] Ray A., Fornsaglio J., Dogan S., Hedau S., Naik D., De A. (2018). Gynaecological cancers and leptin: A focus on the endometrium and ovary. Facts Views Vis. ObGyn.

[87] Taouis M., Benomar Y. (2021). Is resistin the master link between inflammation and inflammation-related chronic diseases?. Mol. Cell Endocrinol..

[88] Nakajima T.E., Yamada Y., Hamano T., Furuta K., Matsuda T., Fujita S., Kato K., Hamaguchi T., Shimada Y. (2010). Adipocytokines as new promising markers of colorectal tumors: Adiponectin for colorectal adenoma, and resistin and visfatin for colorectal cancer. Cancer Sci..

[89] Parafiniuk K., Skiba W., Pawłowska A., Suszczyk D., Maciejczyk A., Wertel I. (2022). The Role of the Adipokine Resistin in the Pathogenesis and Progression of Epithelial Ovarian Cancer. Biomedicines.

[90] Pang S.S., Le Y.Y. (2006). Role of resistin in inflammation and inflammation-related diseases. Cell. Mol. Immunol..

[91] Pang L., Chang X. (2021). Resistin Expression in Epithelial Ovarian Cancer promotes the Proliferation and Migration of Ovarian Cancer Cells to Worsen Prognosis. J. Cancer.

[92] Kukla A., Piotrowska K., Misiek M., Chudecka-Glaz A.M. (2022). Role of adipokines in ovarian cancer epidemiology and prognosis. Ginekol. Polska.

[93] Zhou J., Gong G., Tan H., Dai F., Zhu X., Chen Y., Wang J., Liu Y., Chen P., Wu X. (2015). Urinary microRNA-30a-5p is a potential biomarker for ovarian serous adenocarcinoma. Oncol. Rep..

[94] Ouh Y.T., Cho H.W., Lee J.K., Choi S.H., Choi H.J., Hong J.H. (2019). CXC chemokine ligand 1 mediates adiponectin-induced angiogenesis in ovarian cancer. Tumour Biol..

[95] Beyazit F., Hiz M.M., Turkon H., Unsal M.A. (2021). Serum spexin, adiponectin and leptin levels in polycystic ovarian syndrome in association with FTO gene polymorphism. Ginekol. Pol..

[96] Jin J.H., Kim H.J., Kim C.Y., Kim Y.H., Ju W., Kim S.C. (2016). Association of plasma adiponectin and leptin levels with the development and progression of ovarian cancer. Obstet. Gynecol. Sci..

[97] Słomian G.J., Nowak D., Buczkowska M., Głogowska-Gruszka A., Słomian S.P., Roczniak W., Janyga S., Nowak P. (2019). The role of adiponectin and leptin in the treatment of ovarian cancer patients. Endokrynol. Pol..

[98] Feng Y., Hao F., Wan W., Wang X. (2018). Adiponectin exhibits proliferative and anti-apoptotic effects on ovarian cancer cells via PI3K/Akt and Raf/MEK/ERK pathways. Trop. J. Pharm. Res..

[99] Dakroub A., ANasser S., Younis N., Bhagani H., Al-Dhaheri Y., Pintus G., Eid A.A., El-Yazbi A.F., Eid A.H. (2020). Visfatin: A Possible Role in Cardiovasculo-Metabolic Disorders. Cells.

[100] Gogola-Mruk J., Tworzydło W., Krawczyk K., Marynowicz W., Ptak A. (2023). Visfatin induces ovarian cancer resistance to anoikis by regulating mitochondrial activity. Endocrine.

[101] Nacarelli T., Fukumoto T., Zundell J.A., Fatkhutdinov N., Jean S., Cadungog M.G., Borowsky M.E., Zhang R. (2020). NAMPT Inhibition Suppresses Cancer Stem-like Cells Associated with Therapy-Induced Senescence in Ovarian Cancer. Cancer Res..

[102] Roguska J., Agnieszka Zubkiewicz-Kucharska A. (2018). Chemerin as an early marker of metabolic syndrome. Pediatr. Endocrinol. Diabetes Metab..

[103] Mir M.M., Mir R., Alghamdi M.A.A., Wani J.I., Sabah Z.U., Jeelani M., Marakala V., Sohail S.K., O’haj M., Alharthi M.H. (2022). Differential Association of Selected Adipocytokines, Adiponectin, Leptin, Resistin, Visfatin and Chemerin, with the Pathogenesis and Progression of Type 2 Diabetes Mellitus (T2DM) in the Asir Region of Saudi Arabia: A Case Control Study. J. Pers. Med..

[104] Gao C., Shi J., Zhang J., Li Y., Zhang Y. (2022). Chemerin promotes proliferation and migration of ovarian cancer cells by upregulating expression of PD-L1. J. Zhejiang Univ. Sci. B.

[105] Guzel E.C., Celik C., Abali R., Kucukyalcin V., Celik E., Guzel M., Yilmaz M. (2014). Omentin and chemerin and their association with obesity in women with polycystic ovary syndrome. Gynecol. Endocrinol..

[106] Schmitt M., Gallistl J., Schüler-Toprak S., Fritsch J., Buechler C., Ortmann O., Treeck O. (2022). Anti-Tumoral Effect of Chemerin on Ovarian Cancer Cell Lines Mediated by Activation of Interferon Alpha Response. Cancers.

[107] Kurowska P., Barbe A., Różycka M., Chmielińska J., Dupont J., Rak A. (2018). Apelin in Reproductive Physiology and Pathology of Different Species: A Critical Review. Int. J. Endocrinol..

[108] Hoffmann M., Fiedor E., Ptak A. (2017). Bisphenol A and its derivatives tetrabromobisphenol A and tetrachlorobisphenol A induce apelin expression and secretion in ovarian cancer cells through a peroxisome proliferator-activated receptor gamma-dependent mechanism. Toxicol. Lett..

[109] Neelakantan D., Dogra S., Devapatla B., Jaiprasart P., Mukashyaka M.C., Janknecht R., Dwivedi S.K.D., Bhattacharya R., Husain S., Ding K. (2019). Multifunctional APJ Pathway Promotes Ovarian Cancer Progression and Metastasis. Mol. Cancer Res..

[110] Waluga E., Komosińska-Vassev K., Szczepański J., Olczyk P. (2018). Omentyna—Nowy biomarker w medycynie?. Chem. Klin..

[111] Tahmasebpour N., Hosseinpour Feizi M.A., Ziamajidi N., Pouladi N., Montazeri V., Farhadian M., Abbasalipourkabir R. (2020). Association of Omentin-1 with Oxidative Stress and Clinical Significances in Patients with Breast Cancer. Adv. Pharm. Bull..

[112] Paval D.R., Di Virgilio T.G., Skipworth R.J.E., Gallagher I.J. (2022). The Emerging Role of Intelectin-1 in Cancer. Front. Oncol..

[113] Arjmand M.-H., Moradi A., Akbari A., Mehrad-Majd H. (2020). Clinical Significance of Circulating Omentin Levels in Various Malignant Tumors: Evidence From a Systematic Review and Meta-Analysis. Cytokine.

[114] Au-Yeung C.L., Yeung T.L., Achreja A., Zhao H., Yip K.P., Kwan S.Y., Onstad M., Sheng J., Zhu Y., Baluya D.L. (2020). ITLN1 modulates invasive potential and metabolic reprogramming of ovarian cancer cells in omental microenvironment. Nat. Commun..

[115] Pasquier J., Rafii A. (2013). Role of the microenvironment in ovarian cancer stem cell maintenance. BioMed Res. Int..

[116] Bar J.K., Grelewski P., Lis-Nawara A., Drobnikowska K. (2015). The role of cancer stem cells in progressive growth and resistance of ovarian cancer: True or fiction?. Postep. Hig. Med. Dosw.

[117] Lupia M., Cavallaro U. (2017). Ovarian cancer stem cells: Still an elusive entity?. Mol. Cancer.

[118] Kenda Suster N., Virant-Klun I. (2019). Presence and role of stem cells in ovarian cancer. World J. Stem. Cells.

[119] Tarhriz V., Bandehpour M., Dastmalchi S., Ouladsahebmadarek E., Zarredar H., Eyvazi S. (2019). Overview of CD24 as a new molecular marker in ovarian cancer. J. Cell. Physiol..

[120] Nakamura K., Terai Y., Tanabe A., Ono Y.J., Hayashi M., Maeda K., Fujiwara S., Ashihara K., Nakamura M., Tanaka Y. (2017). CD24 expression is a marker for predicting clinical outcome and regulates the epithelial-mesenchymal transition in ovarian cancer via both the Akt and ERK pathways. Oncol. Rep..

[121] Choi Y.L., Kim S.H., Shin Y.K., Hong Y.C., Lee S.J., Kang S.Y., Ahn G. (2005). Cytoplasmic CD24 expression in advanced ovarian serous borderline tumors. Gynecol. Oncol..

[122] Zhang S., Balch C., Chan M.W., Lai H.C., Matei D., Schilder J.M., Yan P.S., Huang T.H., Nephew K.P. (2008). Identification and characterization of ovarian cancer-initiating cells from primary human tumors. Cancer Res..

[123] Lin J., Ding D. (2017). The prognostic role of the cancer stem cell marker CD44 in ovarian cancer: A meta-analysis. Cancer Cell Int..

[124] Tao Y., Li H., Huang R., Mo D., Zeng T., Fang M., Li M. (2018). Clinicopathological and Prognostic Significance of Cancer Stem Cell Markers in Ovarian Cancer Patients: Evidence from 52 Studies. Cell. Physiol. Biochem..

[125] Zhou J., Du Y., Lu Y., Luan B., Xu C., Yu Y., Zhao H. (2019). CD44 Expression Predicts Prognosis of Ovarian Cancer Patients Through Promoting Epithelial-Mesenchymal Transition (EMT) by Regulating Snail, ZEB1, and Caveolin-1. Front. Oncol..

[126] Stemberger-Papić S., Vrdoljak-Mozetic D., Ostojić D.V., Rubesa-Mihaljević R., Krigtofić I., Brncić-Fisher A., Kragević M., Eminović S. (2015). Expression of CD133 and CD117 in 64 Serous Ovarian Cancer Cases. Coll. Antropol..

[127] Yang B., Yan X., Liu L., Jiang C., Hou S. (2017). Overexpression of the cancer stem cell marker CD117 predicts poor prognosis in epithelial ovarian cancer patients: Evidence from meta-analysis. OncoTargets Ther..

[128] Luo L., Zeng J., Liang B., Zhao Z., Sun L., Cao D., Yang J., Shen K. (2011). Ovarian cancer cells with the CD117 phenotype are highly tumorigenic and are related to chemotherapy outcome. Exp. Mol. Pathol..

[129] Curley M.D., Therrien V.A., Cummings C.L., Sergent P.A., Koulouris C.R., Friel A.M., Roberts D.J., Seiden M.V., Scadden D.T., Rueda B.R. (2009). CD133 expression defines a tumor initiating cell population in primary human ovarian cancer. Stem Cells.

[130] Zhang J., Guo X., Chang D.Y., Rosen D.G., Mercado-Uribe I., Liu J. (2012). CD133 expression associated with poor prognosis in ovarian cancer. Mod. Pathol..

[131] Zhou Q., Chen A., Song H., Tao J., Yang H., Zuo M. (2015). Prognostic value of cancer stem cell marker CD133 in ovarian cancer: A meta-analysis. Int. J. Clin. Exp. Med..

[132] Onisim A., Iancu M., Vlad C., Kubelac P., Fetica B., Fulop A., Achimas-Cadariu A., Achimas-Cadariu P. (2016). Expression of Nestin and CD133 in serous ovarian carcinoma. J. BUON.

[133] Liu B.L., Liu S.J., Baskys A., Cheng H., Han Y., Xie C., Song H., Li J., Xin X.Y. (2014). Platinum sensitivity and CD133 expression as risk and prognostic predictors of central nervous system metastases in patients with epithelial ovarian cancer. BMC Cancer.

[134] Wang Y., Shao F., Chen L. (2018). ALDH1A2 suppresses epithelial ovarian cancer cell proliferation and migration by downregulating STAT3. OncoTargets Ther..

[135] Wang Y.C., Yo Y.T., Lee H.Y., Liao Y.P., Chao T.K., Su P.H., Lai H.C. (2012). ALDH1-bright epithelial ovarian cancer cells are associated with CD44 expression, drug resistance, and poor clinical outcome. Am. J. Pathol..

[136] Landen C.N., Goodman B., Katre A.A., Steg A.D., Nick A.M., Stone R.L., Miller L.D., Mejia P.V., Jennings N.B., Gershenson D.M. (2010). Targeting aldehyde dehydrogenase cancer stem cells in ovarian cancer. Mol. Cancer Ther..

[137] Huang R., Li X., Holm R., Trope C.G., Nesland J.M., Suo Z. (2015). The expression of aldehyde dehydrogenase 1 (ALDH1) in ovarian carcinomas and its clinicopathological associations: A retrospective study. BMC Cancer.

[138] Zhao W., Zang C., Zhang T., Li J., Liu R., Feng F., Lv Q., Zheng L., Tian J., Sun C. (2018). Clinicopathological characteristics and prognostic value of the cancer stem cell marker ALDH1 in ovarian cancer: A meta-analysis. Onco Targets Ther..

[139] Ayub T.H., Keyver-Paik M.D., Debald M., Rostamzadeh B., Thiesler T., Schröder L., Barchet W., Abramian A., Kaiser C., Kristiansen G. (2015). Accumulation of ALDH1-positive cells after neoadjuvant chemotherapy predicts treatment resistance and prognosticates poor outcome in ovarian cancer. Onco Target.

[140] Zhang J., Chang D.Y., Mercado-Uribe I., Liu J. (2012). Sex-determining region Y-box 2 expression predicts poor prognosis in human ovarian carcinoma. Hum. Pathol..

[141] Bååth M., Westbom-Fremer S., Martin de la Fuente L., Ebbesson A., Davis J., Malander S., Måsbäck A., Kannisto P., Hedenfalk I. (2020). SOX2 is a promising predictor of relapse and death in advanced stage high-grade serous ovarian cancer patients with residual disease after debulking surgery. Mol. Cell. Oncol..

[142] Li Y., Chen K., Li L., Li R., Zhang J., Ren W. (2015). Overexpression of SOX2 is involved in paclitaxel resistance of ovarian cancer via the PI3K/Akt pathway. Tumor Biol..

[143] Alshamrani A.A. (2020). Roles of microRNAs in Ovarian Cancer Tumorigenesis: Two Decades Later, What Have We Learned?. Front. Oncol..

[144] Palma Flores C., García-Vázquez R., Gallardo Rincón D., Ruiz-García E., Astudillo de la Vega H., Marchat L.A., Salinas Vera Y.M., López-Camarillo C. (2017). MicroRNAs driving invasion and metastasis in ovarian cancer: Opportunities for translational medicine (Review). Int. J. Oncol..

[145] Lee L.W., Zhang S., Etheridge A., Ma L., Martin D., Galas D., Wang K. (2010). Complexity of the microRNA repertoire revealed by next-generation sequencing. RNA.

[146] Alwani A., Baj-Krzyworzeka M. (2021). miRNAs—Targets in cancer therapy. Postępy Biochem..

[147] Weber J.A., Baxter D.H., Zhang S., Huang D.Y., Huang K.H., Lee M.J., Galas D.J., Wang K. (2010). The microRNA spectrum in 12 body fluids. Clin. Chem..

[148] Kaźmierczak D., Sterzyńska K., Januchowski R. (2021). Role of miRNA in ovarian cancer diagnosis, prognosis and development of drug resistance. Postępy Biol. Komórki Tom.

[149] Resnick K.E., Alder H., Hagan J.P., Richardson D.L., Croce C.M., Cohn D.E. (2009). The detection of differentially expressed microRNAs from the serum of ovarian cancer patients using a novel real-time PCR platform. Gynecol. Oncol..

[150] Nam E.J., Yoon H., Kim S.W., Kim H., Kim Y.T., Kim J.H., Kim J.W., Kim S. (2008). MicroRNA expression profiles in serous ovarian carcinoma. Clin. Cancer Res..

[151] Vilming Elgaaen B., Olstad O.K., Haug K.B., Brusletto B., Sandvik L., Staff A.C., Gautvik K.M., Davidson B. (2014). Global miRNA expression analysis of serous and clear cell ovarian carcinomas identifies differentially expressed miRNAs including miR-200c-3p as a prognostic marker. BMC Cancer.

[152] Chung Y.W., Bae H.S., Song J.Y., Lee J.K., Lee N.W., Kim T., Lee K.W. (2013). Detection of microRNA as novel biomarkers of epithelial ovarian cancer from the serum of ovarian cancer patients. Int. J. Gynecol. Cancer.

[153] Wang S., Zhao X., Wang J., Wen Y., Zhang L., Wang D., Chen H., Chen Q., Xiang W. (2013). Upregulation of microRNA-203 is associated with advanced tumor progression and poor prognosis in epithelial ovarian cancer. Med. Oncol..

[154] Teng Y., Su X., Zhang X., Zhang Y., Li C., Niu W., Liu C., Qu K. (2016). miRNA-200a/c as potential biomarker in epithelial ovarian cancer (EOC): Evidence based on miRNA meta-signature and clinical investigations. Oncotarget.

[155] Zhu Z., Chen Z., Wang M., Zhang M., Chen Y., Yang X., Zhou C., Liu Y., Hong L., Zhang L. (2022). Detection of plasma exosomal miRNA-205 as a biomarker for early diagnosis and an adjuvant indicator of ovarian cancer staging. J. Ovarian Res..

[156] Vang S., Wu H.T., Fischer A., Miller D.H., MacLaughlan S., Douglass E., Comisar L., Steinhoff M., Collins C., Smith P.J. (2013). Identification of ovarian cancer metastatic miRNAs. PLoS ONE.

[157] Nowak E., Wasińska M., Bednarek I. (2017). Epigenetic changes in breast cancerand ovarian cancer—Part II mechanisms of carcinogenesis. Postępy Biol. Komórki Tom.

[158] Zhang H., Lu B. (2020). microRNAs as biomarkers of ovarian cancer. Expert Rev. Anticancer. Ther..

[159] Macdonald I.K., Parsy-Kowalska C.B., Chapman C.J. (2017). Autoantibodies: Opportunities for Early Cancer Detection. Trends Cancer.

[160] Yang W.-L., Simmons A., Lu Z., Baggerly K., Lu K., Gentry-Maharaj A., Menon U., Jacobs I., Bast R.C. (2015). Abstract 2838: TP53 autoantibody can detect CA125 screen negative ovarian cancer cases and can be elevated prior to CA125 in preclinical ovarian cancer. Cancer Res..

[161] Ma Y., Wang X., Qiu C., Qin J., Wang K., Sun G., Jiang D., Li J., Wang L., Shi J. (2021). Using protein microarray to identify and evaluate autoantibodies to tumor-associated antigens in ovarian cancer. Cancer Sci..

[162] Wang P., Qin J., Ye H., Li L., Wang X., Zhang J. (2019). Using a panel of multiple tumor-associated antigens to enhance the autoantibody detection in the immunodiagnosis of ovarian cancer. J. Cell Biochem..

[163] Liu W., de la Torre I.G., Gutiérrez-Rivera M.C., Wang B., Liu Y., Dai L., Qian W., Zhang J.Y. (2015). Detection of autoantibodies to multiple tumor-associated antigens (TAAs) in the immunodiagnosis of breast cancer. Tumor Biol..

[164] Pessoa L.S., Heringer M., Ferrer V.P. (2020). ctDNA as a cancer biomarker: A broad overview. Crit. Rev. Oncol. Hematol..

[165] Lu Y., Li L. (2021). The prognostic value of circulating tumor DNA in ovarian cancer: A meta-analysis. Technol. Cancer Res. Treat..

